# A randomized single-dose, two-period crossover bioequivalence study of two fixed-dose Paracetamol/Orphenadrine combination preparations in healthy volunteers under fasted condition

**DOI:** 10.1186/s40360-020-00416-3

**Published:** 2020-06-23

**Authors:** Kit Yee Cheah, Kar Yee Mah, Lai Hui Pang, Shi Min Ng, Jia Woei Wong, Siew Siew Tan, Hong Zhe Tan, Kah Hay Yuen

**Affiliations:** 1 Clinical Research Ward, Clinical Trial Unit, Institute for Clinical Research, National Institutes of Health, Ministry of Health Malaysia, Hospital Ampang, Ampang, Selangor Malaysia; 2 Pharmacy-Attest Research Sdn Bhd BA/BE Centre, George Town, Pulau Pinang Malaysia

**Keywords:** Bioequivalence, Fasted, Orphenadrine, Paracetamol

## Abstract

**Background:**

Paracetamol/Orphenadrine is a fixed dose combination containing 35 mg orphenadrine and 450 mg paracetamol. It has analgesic and muscle relaxant properties and is widely available as generics. This study is conducted to investigate the relative bioavailability and bioequivalence between one fixed dose paracetamol/orphenadrine combination test preparation and one fixed dose paracetamol/orphenadrine combination reference preparation in healthy volunteers under fasted condition for marketing authorization in Malaysia.

**Method:**

This is a single-center, single-dose, open-label, randomized, 2-treatment, 2-sequence and 2-period crossover study with a washout period of 7 days. Paracetamol/Orphenadrine tablets were administered after a 10-h fast. Blood samples for pharmacokinetic analysis were collected at scheduled time intervals prior to and up to 72 h after dosing. Blood samples were centrifuged, and separated plasma were kept frozen (− 15 °C to − 25 °C) until analysis. Plasma concentrations of orphenadrine and paracetamol were quantified using liquid-chromatography-tandem mass spectrometer using diphenhydramine as internal standard. The pharmacokinetic parameters AUC_0-∞_, AUC_0-t_ and C_max_ were determined using plasma concentration time profile for both preparations. Bioequivalence was assessed according to the ASEAN guideline acceptance criteria for bioequivalence which is the 90% confidence intervals of AUC_0-∞_, AUC_0-t_ and C_max_ ratio must be within the range of 80.00–125.00%.

**Results:**

There were 28 healthy subjects enrolled, and 27 subjects completed this trial. There were no significant differences observed between the AUC_0-∞_, AUC_0-t_ and C_max_ of both test and reference preparations in fasted condition. The 90% confidence intervals for the ratio of AUC_0-t_ (100.92–111.27%), AUC_0-∞_ (96.94–108.08%) and C_max_ (100.11–112.50%) for orphenadrine (*n* = 25); and AUC_0-t_ (94.29–101.83%), AUC_0-∞_ (94.77–101.68%) and C_max_ (87.12–101.20%) for paracetamol (*n* = 27) for test preparation over reference preparation were all within acceptable bioequivalence range of 80.00–125.00%.

**Conclusion:**

The test preparation is bioequivalent to the reference preparation and can be used interchangeably.

**Trial registration:**

NMRR- 17-1266-36,001; registered and approved on 12 September 2017.

## Background

**Fig. 1:**
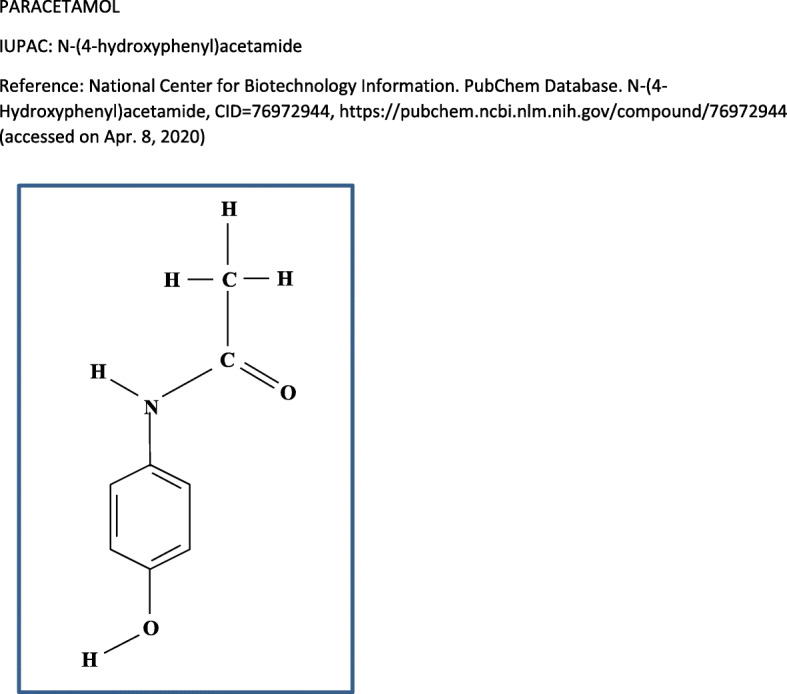
Chemical structure of paracetamol [1]

One of the most well-known and commonly used analgesics in the world is paracetamol. Paracetamol is a para-aminophenol derivative, with antipyretic and weak anti-inflammatory activity. Its IUPAC name is *N*-(4-hydroxyphenyl) acetamide and chemical structure is as displayed in Fig. 1. Its’ mechanism of action is still not well-known despite its widespread use, but is thought to act through a combination of related pathways involving an inhibition of prostaglandin production and the nitric oxide pathway [2, 3]. Paracetamol is absorbed mainly from the small bowel, and exerts analgesic effect within 40 min, with a variable t_max_ of 10 min to 1 h, due to large variation in bioavailability (63–89% for oral) [3]. About 80% of paracetamol is excreted in the urine after conjugation and it has a half-life of 1–4 h [4]. Paracetamol can be used alone or as an adjunct with other drugs to produce a synergistic effect. One of these drugs is orphenadrine, where it is used to provide an added analgesic effect in addition to muscle relaxant in the treatment of painful muscle spasms.

**Fig. 2:**
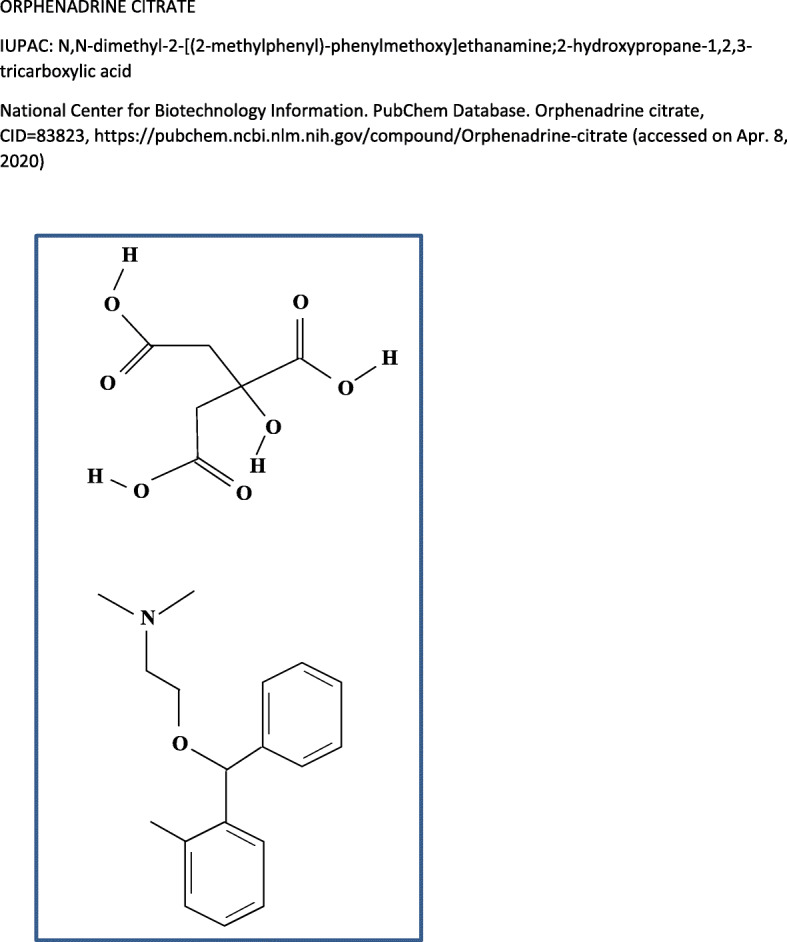
Chemical structure of orphenadrine [5]

Orphenadrine is an anticholinergic drug belonging to the ethanolamine antihistamine class; it is a derivative of diphenhydramine. It has an IUPAC name of (RS)-N,N-Dimethyl-2-[(2-methylphenyl)-phenyl-methoxy]-ethanamine and a chemical structure as displayed in Fig. 2. It is classed as a skeletal muscle relaxant drug and is thought to act by blocking muscarinic acetylcholine receptors and N-methyl-D-aspartate receptors in the central nervous system, hence producing a muscle relaxant effect by affecting the transmission of nerve impulses between the spinal cord and muscles [6]. As a diphenhydramine analogue, orphenadrine can also produce some antihistaminic effects and local anesthetic effects. Orphenadrine is readily absorbed from the gastrointestinal tract and takes up to 1 h to exert an effect after oral administration [6, 7]. It has a half-life of approximately 14 h and undergoes biotransformation in the liver to the pharmacologically active metabolites N-demethyl orphenadrine and N,N-didemethyl orphenadrine [6].

A few clinical trials investigating the effect of a combination of orphenadrine and paracetamol tablets on myalgia demonstrated an increased pain relief effect as compared to just placebo [8, 9]. A review on the efficacy of a combination of orphenadrine and paracetamol also found a few well-designed studies that supported the increased efficacy of orphenadrine/paracetamol combination as compared to paracetamol alone [10]. Orphenadrine and paracetamol co-administered as a fixed dose combination drug is used to treat tension headache, occipital headaches associated with spasms of skeletal muscles in the region of the head and neck, acute and traumatic conditions of the limbs and trunk such as sprains, strains, whiplash injuries, acute torticollis and prolapsed intervertebral disc [11]. The safety profile of both paracetamol and orphenadrine generally do not produce much adverse effects at their recommended dosages; those that commonly do occur are related to the anticholinergic property of orphenadrine which includes dryness of mouth, tachycardia, palpitation, urinary hesitancy or retention, blurred vision, dilation of pupils, increased ocular tension, weakness, nausea, headache, dizziness, constipation and drowsiness [11]. Less common side effects include rash and sedation, but these effects disappear upon discontinuation of the drug.

The fixed dose combination of orphenadrine/paracetamol is available as both an innovator and a generic in Malaysia. Currently in Malaysia, there are at least 7 different brands of fixed dose combincation orphenadrine/paracetamol such as Norgesic, Dycosic, Anarex, Orphenamol, Myoflex, Suniton and Norphenadol tablet as registered on https://npra.gov.my/index.php/en/. With the increasing availability of generic drugs, during the 236th Drug Control Authority (DCA) meeting in 2012, the Malaysian government has decided to include bioequivalence (BE) studies as a requirement for new generic drug registration [12]. There is also a lack of literature on the bioequivalence of paracetamol/orphenadrine combination globally as well, hence such a clinical trial can increase the knowledge in this area in addition to being a precursor for generic drug registration. The aim of the current study was to compare the rate and extent of absorption of orphenadrine from a test preparation to an equivalent oral dose of a reference preparation in heathy volunteers under fasted condition.

## Methods

### Study design

This was a single-center, single-dose, open-label, randomized, 2-treatment, 2-sequence, 2-period crossover bioequivalence study that was conducted in 3 different sites in Malaysia. The clinical phase was conducted at Clinical Research Ward (CRW), Hospital Ampang; the bioanalysis was done at BA/BE Laboratory, Attest Research Sdn Bhd and the statistical analysis was evaluated at Pharmacy-Attest Research Sdn. Bhd, School of Pharmaceutical Sciences.

Subjects underwent a health screening within 30 days before the start of the first study period, to ensure their healthy status was valid before the start of the first study period. For each study period, they were admitted to the study unit at least 11 h before study medication administration and were discharged after the 24 h post-dose blood sample was collected. This was done to ensure that subjects were housed in a controlled environment during the critical blood sampling stage. Subjects returned to the unit for two ambulatory visits at 48- and 72-h post-dose of each study period. In each study period, subjects received a single dose of either test preparation (T) (hovid-Horgesic tablet, manufactured by Hovid Bhd) or reference preparation (R) (Norgesic tablet, manufactured by Adcock Ingram Ltd. – A Medreich Group Company). Each tablet contained 35 mg orphenadrine citrate and 450 mg paracetamol. Study medication was stored in a temperature-controlled pharmacy at the clinical site until dosing.

The two study periods were separated by a 7-day washout, which was longer than the minimal of 10 orphenadrine elimination half-lives to ensure no carry-over effects. All subjects underwent an overnight fast of at least 10 h with free access of plain water. Study medication were administered orally with 240 ml of plain water at room temperature in the morning and a mouth check was done with flashlight assistance to ensure compliance to study medication. For each study period, subjects adhered to restrictions where they were not allowed any water 1 h before and 1 h after study medication administration except those used for drug administration. They remained fasted for at least 4 h after dosing. Standardized meals were served at around 4.17 h and 11.25 h after dosing and snacks were served at 7 h and 13 h. The same meals were served for both study periods. Subjects were also not allowed to smoke during their entire stay at the study unit. Subjects’ safety was monitored throughout the study where their vital signs which include blood pressure, pulse, body temperature and respiratory rate were recorded prior to the admission into study unit and were conducted again at approximately 2, 6, 10, 14, 24 and 72 h after dosing. All adverse events were evaluated by medical doctors.

Blood samples of 8 ml volume were collected in vacutainers containing sodium heparin as an anticoagulant, at time intervals of 0.00 (pre-dose), 0.33, 0.66, 1.00, 1.50, 2.00, 2.33, 2.66, 3.00, 3.33, 3.66, 4.00, 5.00, 8.00, 12.00, 16.00, 24.00, 48.00 and 72.00 h after dosing. With the exception of the pre-dose and ambulatory blood samples which were collected via direct venipuncture, all other samples were collected via an in-dwelling catheter. One ml of sodium heparin was used to flush the catheter prior to blood collection to maintain catheter patency. Samples were centrifuged for 15 min at 3500 rpm and 25 °C immediately upon collection. The obtained plasma samples were transferred to cryovials and kept frozen between − 15 °C to − 25 °C until analysis.

### Study population

Healthy male volunteers aged between 18 to 55 years with a body mass index of 18.5 to 29.9 kg/m^2^ were recruited. Subjects were medically healthy, as determined by the investigators at the screening evaluation, with clinically normal ECGs, laboratory tests and with no history of alcohol or drug dependence. Subjects who had a history or presence of organ dysfunction, clinically significant bone-marrow depression, serious blood disorders, cardiac arrhythmias, cardiovascular disease, stroke, bronchospasm, asthma, anaphylaxis, diabetes mellitus, renal diseases, liver diseases, thyrotoxicosis, parkinsonism, benign prostatic hypertrophy, epilepsy or migraine and malignancy, or consumption of drugs that may alter gastrointestinal motility 1 week prior to admission were excluded. Informed consent was obtained from all volunteers prior to the start of study.

The number of subjects recruited into the study was estimated using the intrasubject coefficient of variation (ISCV) [13]. However, as there is little information about the intrasubject CV of fixed dose combination of orphenadrine and paracetamol in bioequivalence study to date, a total of 28 subjects were enrolled for this study with the assumption that the ISCV of will not be more than 20% and to account for dropouts. This sample size fulfilled the requirements of ASEAN Guideline for The Conduct of Bioequivalence Studies (2015) and European Medicines Agency (2010) [14, 15]. The power of the study was calculated after completion of the study.

The study was approved by the Medical Research & Ethics Committee (MREC), Ministry of Health Malaysia. This study was carried out in accordance to the current ICH Guidelines on Good Clinical Practice (GCP) and Declaration of Helsinki [16, 17]. The writing of this manuscript also adheres to the CONSORT guidelines.

### Randomization

Subjects were randomized equally into one block size, by using software available from http://www.randomization.com, into 2 groups to receive test preparation (T) and reference preparation (R) according to the following sequences: TR or RT. The principal investigator generated the randomization schedule before commencement of the study and the clinical site enrolled the subjects sequentially on a first-come-first-serve basis. They were then allocated to their groups according to the randomization schedule. The bioanalytical site was kept blinded to the randomization schedule until the completion of the clinical and analytical phases.

### Sample analysis

Analysis of plasma orphenadrine and paracetamol concentration was conducted according to European Medicines Agency – Guideline on Bioanalytical Method Validation (EMA, 2011) [18]. Plasma levels of orphenadrine and paracetamol were analyzed using liquid-chromatography-tandem mass spectrometer (LCMSMS). The current assay method was developed based on the LC-MS/MS method by Lee et al. [19]. The LC system comprised an Agilent 1200 Series binary pump (Agilent, Waldbronn, Germany), an Agilent 1200 Series degasser (Agilent, Waldbronn, Germany), an Agilent 1200 Series thermostated column compartment (Agilent, Waldbronn, Germany) and an Agilent 1200 Series instant pilot (Agilent, Waldbronn, Germany). MS/MS analyses were performed on an Applied Biosystems API 3200 triple quadrupole mass spectrometer (Applied Biosystems/MDS SCIEX, Ontario, Canada) in positive electrospray ionization (ESI) mode. Data acquisition and analysis were performed using the software Analyst version 1.4.2 (Applied Biosystems/MDS SCIEX, Ontario, Canada).

**Fig. 3:**
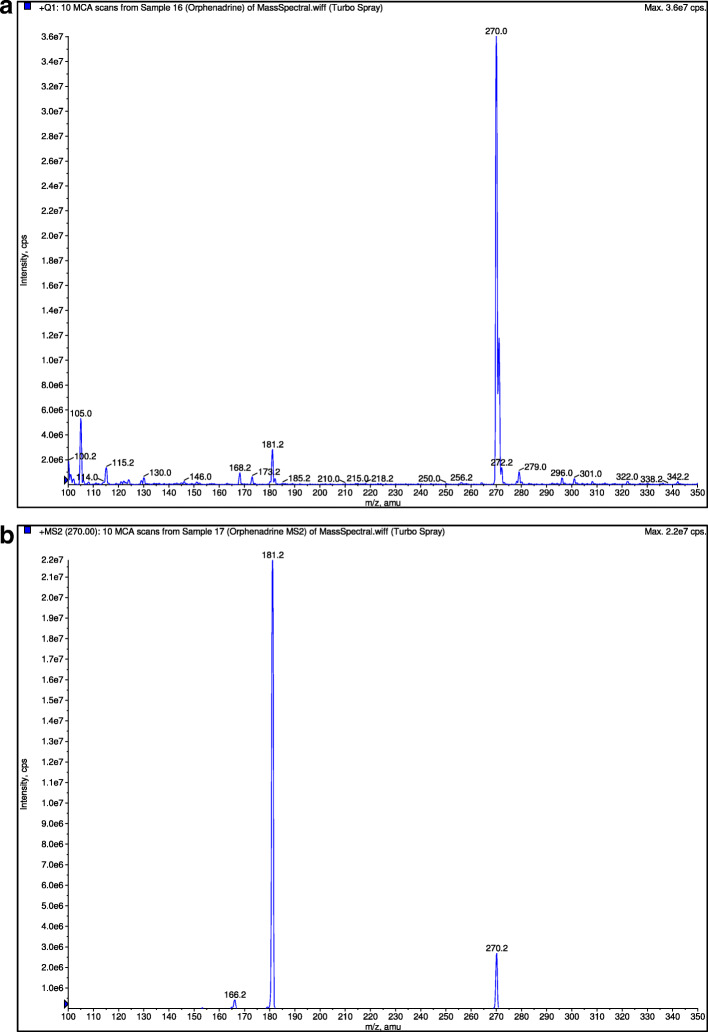
**a** Mass spectra of orphenadrine. **b** Mass spectra of orphenadrine

**Fig. 4:**
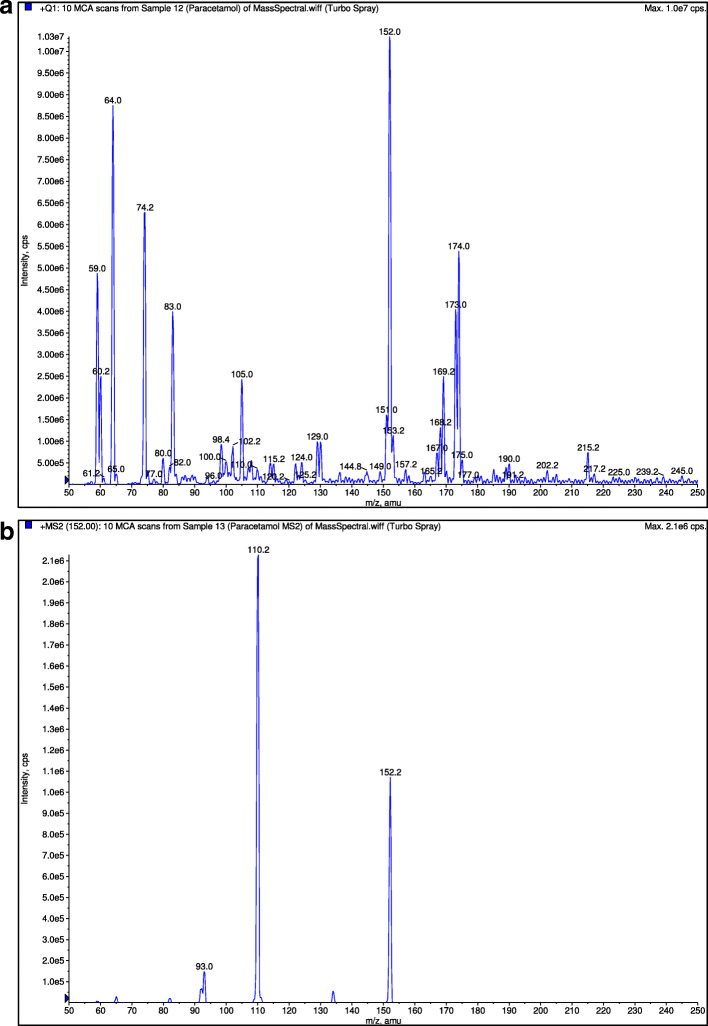
**a** Mass spectra of paracetamol. **b** Mass spectra of paracetamol

**Fig. 5:**
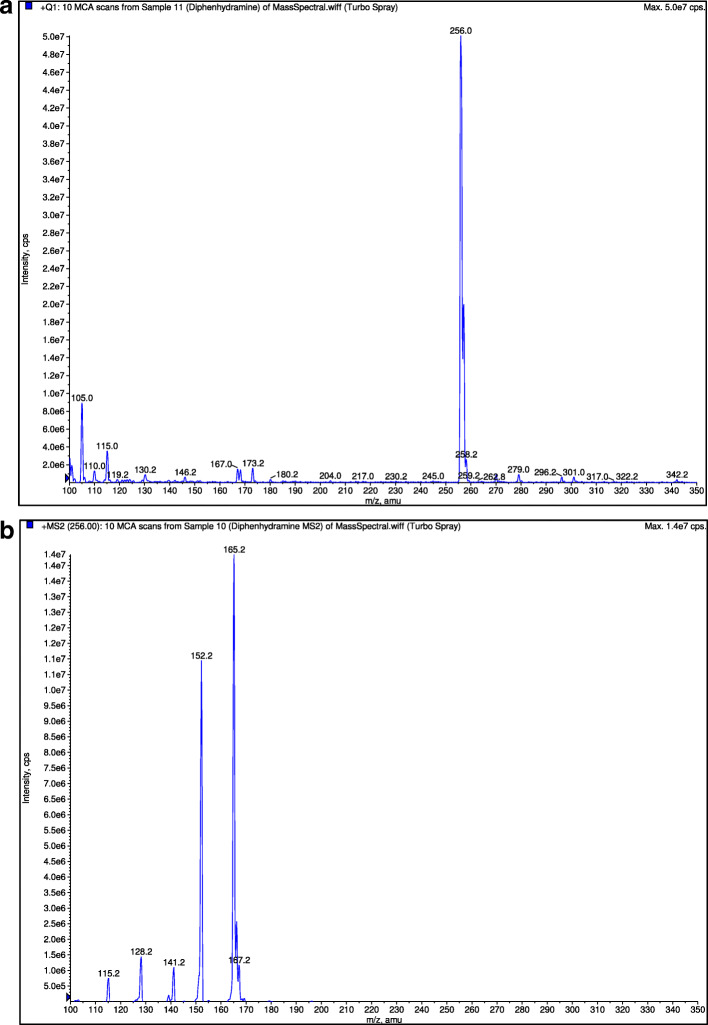
**a** Mass spectra of diphenhydramine. **b** Mass spectra of diphenhydramine

A C18 column (150 mm × 2 mm, 3 μm) analytical column at ambient room temperature was used for the chromatographic separation. The mobile phase consists of a mixture of 50% methanol, 30% acetonitrile, and 20% 0.01 M ammonium formate with the pH adjusted to 3.0 with formic acid. Analysis was performed isocratically at a flow rate of 0.15 ml/min. The retention times of orphenadrine, paracetamol and diphenhydramine (internal standard) were around 2.8 min, 2.7 min and 2.7 min respectively. The mass transition for orphenadrine, paracetamol and diphenhydramine were detected at multiple reactions monitoring mode at m/z 270.0 → 181.0 (Fig. 3), m/z 152.0 → 110.0 (Fig. 4) and m/z 256.0 → 152.0 (Fig. 5), respectively. The detector response for orphenadrine and paracetamol were linear over a concentration range of 2–80 ng/ml for orphenadrine and 200–8000 ng/ml for paracetamol with correlation coefficient of at least 0.99. The lower limit of quantification (LLOQ) was set at 2 ng/ml for orphenadrine and 200 ng/ml for paracetamol with a signal to noise ratio of at least 5:1 for both analytes. Quality control samples (QC samples) were injected intermittently during each day of analysis of samples obtained from the bioequivalence study. The within-day and between-day accuracy of the bioanalytical method for orphenadrine was 93.2–110% and 93.5–107.0%, respectively. The within-day and between-day accuracy of the bioanalytical method for paracetamol was 86.4–96.8% and 86.0% - 114.3, respectively. As for the precision, the within-day and between-day coefficient of variation (CV) values for orphenadrine were < 10.0 and < 13.8%, respectively while at LLOQ, the CV was less than 20%. The respective CV values obtained by paracetamol for within-day and between-day validation were < 5.7% and < 6.2%. The method was deemed selective as no significant endogenous peak was found from eight different sources of blank plasma at the retention times of orphenadrine, paracetamol and diphenhydramine (internal standard). Both the orphenadrine and paracetamol samples were stable for 7 h when stored at room temperature and were still stable after six freeze and thaw cycles. They also had a long-term stability of up to 40 days when stored in cryovial under frozen condition.

**Fig. 6:**
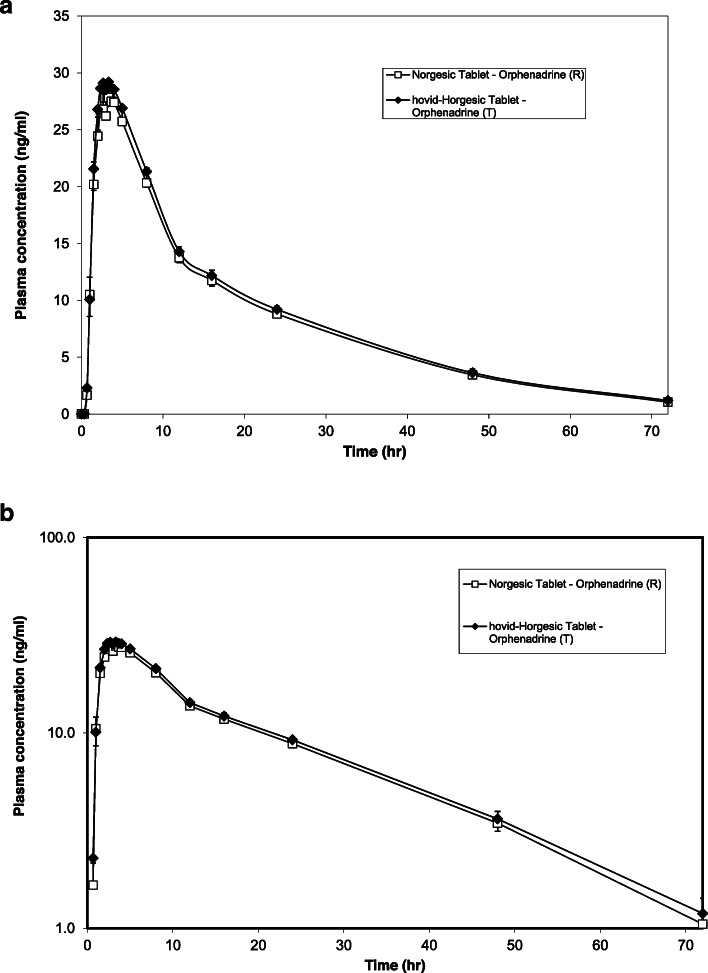
**a** Geometric mean orphenadrine plasma concentration vs. time in linear scale following the administration of the test and reference products. *Error bars* represent the standard error of the mean. (*n* = 25). **b** Geometric mean orphenadrine plasma concentration vs. time in semi-logarithmic scale following the administration of the test and reference products. *Error bars* represent the standard error of the mean. (*n* = 25)

### Pharmacokinetic and statistical analysis

The pharmacokinetic parameters for orphenadrine and paracetamol: maximum plasma concentration (C_max_), time to reach maximum plasma concentration (t_max_), area under the plasma concentration-time curve from time zero to the last measurable concentration (AUC_0-t_) and total area under the plasma concentration-time curve (AUC_0-∞_) were estimated from the plasma concentration-time data. Calculation of these pharmacokinetic parameters was conducted using PhoenixTM WinNonlin 6.4 from CertaraTM, USA. Other pharmacokinetic parameters evaluated were elimination rate constant (K_e_) and half-life (t_1/2_).

The test and reference preparations were considered bioequivalent if the 90% confidence intervals (CIs) for ratio of C_max_, AUC_0-t_ and AUC_0-∞_ values of orphenadrine in test over reference preparation were within the pre-specified comparability bounds of 80.00–125.00% (transformed values) based on the US FDA (2001). The European Medicines Agency (2010) and ASEAN Guidelines for the Conduct of Bioavailabilty and Bioequivalence Studies (2015) also stipulate a similar range for C_max_, AUC_0-t_ and AUC_0-∞_ [20–23].

The statistical analysis was conducted using the commercial software, PhoenixTM WinNonlin 6.4 from CertaraTM, USA except t_max_ which was analyzed using EquivTestPK from Statistical Solution (Cork, Ireland). Log-transformed values (In) of C_max_, AUC_0-t_ and AUC_0-∞_ and values of K_e_ and t_1/2_ obtained with the two preparations were analyzed using an analysis of variance (ANOVA) procedure which distinguishes effects due to subjects, periods and treatment (Wagner, 1975). On the other hand, the t_max_ values were analyzed using Wilcoxon Signed Rank Test for paired samples.

## Results

Twenty eight healthy volunteers were included in the study with mean (SD) age, 24 (5) years; mean (SD) body weight, 69.9 (10.6) kg and mean (SD) height, 169.1 (5.8) cm. Only 27 subjects completed the study and one subject was withdrawn due to adverse event. The statistical analysis for orphenadrine was performed with only 25 subjects. Two subjects were excluded because their pre-dose sample exceeded the 5% of their C_max_ values. All 27 subjects were included into the statistical analysis for paracetamol. All subjects were screened for recruitment during the 30 days before commencement of the first study period and were followed up for safety assessment up to 2 weeks after the end of the second study period.

**Fig. 7:**
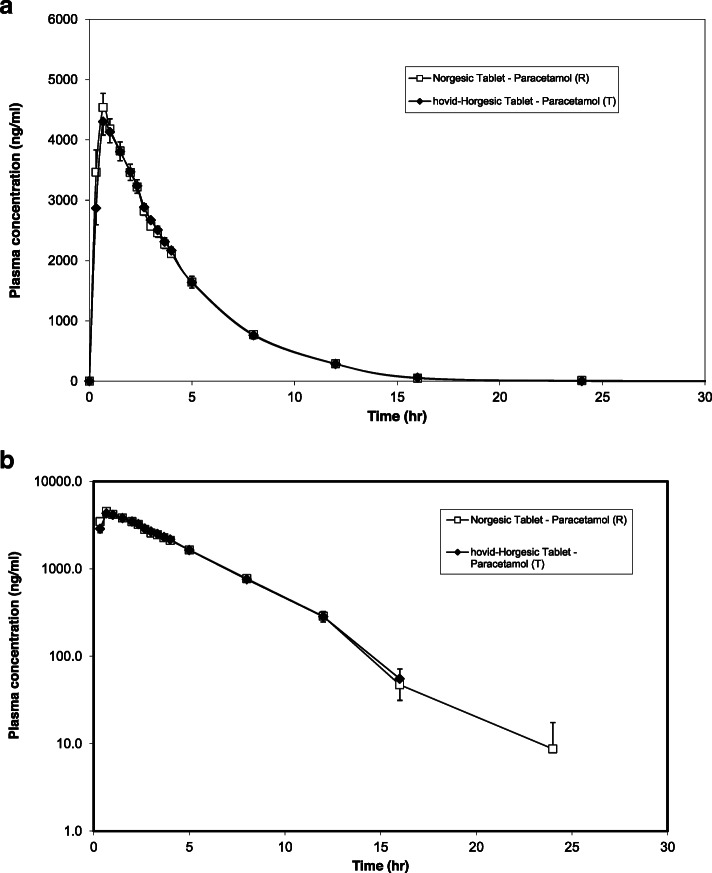
**a** Geometric mean paracetamol plasma concentration vs. time in linear scale following the administration of the test and reference products. *Error bars* represent the standard error of the mean. (*n* = 27). **b** Geometric mean paracetamol plasma concentration vs. time in semi-logarithmic scale following the administration of the test and reference products. *Error bars* represent the standard error of the mean. (*n* = 27)

Figure 6 shows the mean plasma orphenadrine versus time profiles obtained with hovid-Horgesic tablet and Norgesic tablet while Fig. 7 shows the mean plasma paracetamol concentration versus time profiles obtained with both preparations. Overall, the plasma profile in Fig. 6 showed the peak plasma orphenadrine concentrations were attained at approximately 3.33 h while the peak plasma paracetamol concentrations were attained at approximately 0.67 h as showed in Fig. 7.

Table 1 shows the demographic characteristics of the subjects according to either RT or TR group. All the subjects were of Malay ethnicity. The pharmacokinetic properties of orphenadrine from both preparations are summarized in Table 2. Table 3 shows the pharmacokinetic properties of paracetamol from both preparations.

**Table 1:** Demographic characteristics of study subjects of the groups of treatment

Characteristic	RT sequence *N* = 14	TR Sequence *N* = 14	Total *N* = 28
Age (years)	25.43 ± 5.27 (20–41)	23.21 ± 3.76 (20–34)	24.32 ± 4.80 (20–41)
Weight (kg)	70.47 ± 10.66 (53.8–90.2)	69.36 ± 10.11 (58.2–95.9)	69.91 ± 10.59 (53.8–95.9)
Height (cm)	168.79 ± 4.63 (160–179)	169.32 ± 6.62 (157.5–183.5)	169.05 ± 5.83 (157.5–183.5)
Body Mass Index (kg/m^2^)	24.69 ± 3.19 (19.8–29.7)	24.09 ± 2.07 (21.8–28.5)	24.39 ± 2.75 (19.8–29.7)

Data are reported as mean ± standard deviation (range)

**Table 2:** Pharmacokinetic properties of orphenadrine from test and reference preparations (n = 25)

	Mean ± SD (*n* = 25) (Untransformed Data)				CV (%)	
Parameter	Test		Reference		Test	Reference
C_max_ (ng/ml)	32.75 ± 8.05		30.81 ± 7.57		24.60	24.58
t_max_ (hr)	3.39 ± 1.33		3.28 ± 0.91		39.24	27.78
AUC_0-t_ (hr.ng/ml)	572.74 ± 231.43		541.10 ± 233.41		40.41	43.14
AUC_0-∞_ (hr.ng/ml)	671.67 ± 262.47		655.28 ± 273.77		39.08	41.78
K_e_ (hr^-1^)	0.04 ± 0.02		0.04 ± 0.01		37.98	28.15
t_1/2_ (hr)	19.05 ± 6.42		20.69 ± 6.95		33.71	33.61
	Geometric Mean		Test vs. Reference			
				90% CI		
	Test	Reference	Ratio (%)	Lower	Upper	ISCV (%)
C_max_ (ng/ml)	31.76	30.01	105.83	100.11	112.50	12.00
AUC_0-t_ (hr.ng/ml)	527.38	494.81	106.63	100.92	111.27	10.03
AUC_0-∞_ (hr.ng/ml)	623.74	607.28	102.71	96.94	108.08	11.17

*SD* Standard deviation, *CV* Coefficient variation, *C*_*max*_ maximum observed plasma concentration, *t*_*max*_ time to maximum plasma concentration, *AUC*_*0-t*_ Area under the concentration-time curve from the time zero to the time point of last quantifiable plasma concentration, *AUC*_*0-∞*,_ Area under the concentration-time curve from time zero and extrapolated to infinity, *K*_*e*,_ Elimination rate constant, *t*_*1/2*,_ Elimination half-life, *CI* Confidence interval, *ISCV* Intrasubject coefficient variation

**Table 3:** Pharmacokinetic properties of paracetamol from test and reference preparations (N = 27)

	Mean ± SD (*n* = 25) (Untransformed Data)				CV (%)	
Parameter	Test		Reference		Test	Reference
C_max_ (ng/ml)	4616.78 ± 988.83		4917.37 ± 1068.30		21.42	21.73
t_max_ (hr)	0.95 ± 0.49		0.74 ± 0.42		51.53	56.08
AUC_0-t_ (hr.ng/ml)	20062.25 ± 5642.02		20389.03 ± 5666.04		28.12	27.79
AUC_0-∞_ (hr.ng/ml)	21423.27 ± 5858.48		21737.66 ± 5849.22		27.35	26.91
K_e_ (hr^-1^)	0.25 ± 0.05		0.25 ± 0.05		18.69	19.09
t_1/2_ (hr)	2.87 ± 0.55		2.95 ± 0.78		19.08	26.38
	Geometric Mean		Test vs. Reference			
				90% CI		
	Test	Reference	Ratio (%)	Lower	Upper	ISCV (%)
C_max_ (ng/ml)	4515.53	4811.62	93.85	87.12	101.20	16.20
AUC_0-t_ (hr.ng/ml)	19353.20	19754.60	97.97	94.29	101.83	8.26
AUC_0-∞_ (hr.ng/ml)	20716.05	21108.97	98.14	94.77	101.68	7.56

*SD* Standard deviation, *CV* Coefficient variation, *C*_*max*_ maximum observed plasma concentration, *t*_*max,*_ time to maximum plasma concentration, *AUC*_*0-t*,_ area under the concentration-time curve from the time zero to the time point of last quantifiable plasma concentration, *AUC*_*0-∞*,_ Area under the concentration-time curve from time zero and extrapolated to infinity, *K*_*e*,_ elimination rate constant, *t*_*1/2*,_ elimination half-life, *CI* Confidence interval, *ISCV* Intrasubject coefficient variation

For both preparations, the 90% confidence intervals for ratio of C_max_, AUC_0-t_ and AUC_0-∞_ of test preparation over reference preparation were all within the acceptable bioequivalence limit of 80.00 to 125.00% (Tables 2 and 3).

The t_max_ which was calculated using the Wilcoxon Signed Rank test showed no statistically significant differences between the two preparations (*p* = 0.8188 for orphenadrine and *p* = 0.0646 for paracetamol). The median t_max_ of orphenadrine in both preparations is 3.00 h, whereas the median t_max_ of paracetamol in both preparations is 0.67 h.

No statistically significant differences were observed between the mean C_max_ of the two products for orphenadrine (32.75 ng/ml vs 30.81 ng/ml; *p* = 0.1070) and paracetamol (4616.78 ng/ml vs 4917.37 ng/ml; *p* = 0.1597). The t_max_ of orphenadrine for the test product did not differ significantly with that of the reference product (3.39 h vs 3.28 h; *p* = 0.6366) whereas the t_max_ of paracetamol of the two preparations also showed no statistically significant difference as well (0.95 h vs 0.74 h; *p* = 0.0646).

The t_1/2_ of orphenadrine of the two preparations had no statistically significant differences with each other (19.05 h vs 20.69 h; *p* = 0.1853) whereas the t_1/2_ of paracetamol was not statistically different between the two preparations (2.87 h vs 2.95 h; *p* = 0.4428).

## Discussion

As of 2015, Malaysia has adopted the ASEAN Guidelines for the Conduct of Bioequivalence Studies which supersedes the Malaysian Guidelines for the Conduct of Bioavailability and Bioequivalence Studies (2000) to guide the conduct of bioequivalence studies by Sponsors of generic drugs [13]. As per the ASEAN Guideline for the Conduct of Bioequivalence Studies (2015) [13], which is based on the EMA Guideline on Investigation of Bioequivalence (2010) [14], two medicinal products are bioequivalent when their 90% confidence interval (CI) of the AUC_0-t_, AUC_0-∞_ and C_max_ of the generic preparation over the innovator preparation (ln transformed values) fall between the pre-determined limits of 80–125%. In this study, bioequivalence was demonstrated between both the generic and innovator fixed dose combination of paracetamol/orphenadrine as the 90% CIs of the AUC_0-t_, AUC_0-∞_ and C_max_ of orphenadrine are within the pre-specified limits of 80–125% (Tables 1 and 2). The geometric mean ratios of the PK parameters were close to 100% as well. The bioequivalence of the two preparations does not take into account the 90% CIs for ratio of C_max_, AUC_0-t_ and AUC_0-∞_ values of paracetamol as paracetamol is an over-the-counter (OTC) product and was not considered necessary by the regulatory agency to prove bioequivalence for the purpose of drug registration at the time this study was conducted. Although the analysis of paracetamol was not used for conclusion of bioequivalence, the 90% CIs of AUC_0-t_, AUC_0-∞_ and C_max_ for paracetamol also fall within the pre-specified limits of 80–125%, deigning the two fixed dose preparations to be interchangeable with each other.

The National Pharmaceutical Regulatory Agency (NPRA) which is the Malaysia’s drug regulatory authority maintains a Bioequivalence Centre Compliance Programme, which contains a list of clinical and analytical sites that have been inspected by NPRA. Companies who wish to obtain NPRA approval before marketing their generic drugs in Malaysia need to submit a BE study which has been conducted at sites which are listed in the programme. This is to ensure that all BE studies conducted are according to Good Clinical Practice and Good Laboratory Practice standards. The clinical and bioanalytical sites where this study was conducted at are both listed in the Bioequivalence Centre Compliance Programme by NPRA and the data produced are thus of good quality and obtained ethically. The NPRA also has a list of comparator products to be used as reference products in bioequivalence studies required for drug registration; in this case, Norgesic tablet is listed as the comparator product for fixed dose combination orphenadrine/paracetamol reference, hence the selection of Norgesic tablet as the reference product is also appropriate for this study as a precursor for drug registration.

According to the EMA and ASEAN guidelines on bioequivalence, the conduct of BE studies under fasted condition is the most sensitive condition to detect potential differences between two preparations, hence this study is also well-designed as the subjects had undergone a fast of at least 10 h before dosing of the study medication. The washout period of 7 days is at least a minimum of 10 half lives of orphenadrine; however 2 subjects had a pre-dose sample of more than 5% of their C_max_ value and were thus excluded from the statistical analysis of orphenadrine. These 2 subjects had a half-life of more than 14 h, hence the washout period may not be long enough to exclude carry-over effects of the investigational drug. However, paracetamol was analyzed with all 27 subjects included as no peaks were detected in their pre-dose samples.

No serious adverse events were reported; 7 subjects suffered from 8 adverse events but only 1 event (giddiness) was suspected to be drug-related due to the anticholinergic effect of orphenadrine. Only one subject dropped out and was not admitted for the second study period due to having a viral fever before the admission. Other adverse events which included abrasion due to motor vehicle accident, laceration wound, fracture, back pain, sore throat and mild blood loss due to dislodge of branula were not related to the study drug. Both preparations were well tolerated.

The intra-subject variation of the orphenadrine PK parameters were all lower than 15% (Table 1). This indicates a low variability amongst the Malaysian population on orphenadrine. The power of the AUC_0-t_, AUC_0-∞_ and C_max_ calculated for orphenadrine was 100%. This means that future bioequivalent studies can be conducted with fewer subjects to obtain a minimal study power of at least 80% to fulfil the bioequivalence guidelines [14, 15].

## Conclusion

The fixed-dose combination of orphenadrine and paracetamol preparation is not commonly found in developed countries, hence the lack of literature on bioequivalence studies between these two products [23]. However, orphenadrine/paracetamol combination is very useful to treat viral symptoms of myalgia with anti-pyretic benefits which is more prevalent in tropical countries [24]. Hence, the market of generic orphenadrine/paracetamol combination is important to increase the ease of access of affordable medications. This study found that the generic and innovator products is bioequivalent and can be used interchangeably with each other.

## Data Availability

The data that support the findings of this study are available from the Sponsor, Hovid Bhd but restrictions apply to the availability of these data, which were used under license for the current study, and so are not publicly available. Data are however available from the authors upon reasonable request and with permission of Hovid Bhd.

## Abbreviations

ASEAN: Association of Southeast Asian Nations
AUC_0-∞_: Area under the concentration-time curve from time zero and extrapolated to infinity
AUC_0-t_: Area under the concentration-time curve from time zero to the time point of last quantifiable plasma concentration
BA/BE: Bioavailability/ Bioequivalence
BE: Bioequivalence
CI: Confidence interval
C_max_: Maximum observed plasma concentration
CRW: Clinical Research Ward
CV: Coefficient variation
DCA: Drug Control Authority
ECG: Electrocardiogram
EMA: European Medicines Agency
GCP: Good Clinical Practice
hr: hour
ICH: International Conference on Harmonisation
In: Natural logarithm
ISCV: Intrasubject coefficient variation
K_e_: Elimimation rate constant
LC: Liquid-chromatography
LCMSMS: Liquid-chromatography-tandem mass spectrometer
LLOQ: Lower limit of quantification
M: Molar (moles/litre)
mg: milligram
ml: millilitre
MREC: Medical Research & Ethics Committee
MRM: Multiple reaction monitoring
m/z: mass-to-charge-ratio
n: sample size
NMRR: National Medical Research Register
NPRA: National Pharmaceutical Regulatory Agency
OTC: Over-the-counter
p: Calculated probability
PK: Pharmacokinetic
QC: Quality control
SD: Standard of deviation
T: Test
t_1/2_: Elimination half life
t_max_: Time to reach the maximum plasma concentration
R: Reference
USM: University Sains Malaysia
μl: microlitre
